# The Dysregulation and Prognostic Analysis of STRIPAK Complex Across Cancers

**DOI:** 10.3389/fcell.2020.00625

**Published:** 2020-07-10

**Authors:** Ruiling Xie, Feng Wen, Yong Qin

**Affiliations:** Department of Otolaryngology, Head and Neck Surgery, Peking University First Hospital, Beijing, China

**Keywords:** STRIPAK, bioinformatical analysis, cancer, prognosis, PPP2R1A, TRAF3IP3

## Abstract

The striatin-interacting phosphatase and kinase (STRIPAK) is the highly conserved complex, which gains increased attention in physiology and pathology process recently. However, limited studies reported the details of STRIPAK complex in cancers while some results strongly suggested it plays a vital role in tumorigenesis. Hence, we systematically analyzed the molecular and survival profiles of 18 STRIPAK genes to assess the value of STRIPAK complex across cancers. Our findings revealed the low frequencies of DNA aberrances and incomparable expression difference of STRIPAK genes between normal and tumor tissues, but they showed strong prognostic value in cancers, especially the liver hepatocellular carcinoma (LIHC) and kidney renal clear cell carcinoma (KIRC). Interestingly, STRIPAK genes were observed the opposite pattern of survival and expression in the above two cancer types. PPP2R1A and TRAF3IP3 were proposed as the oncogenic genes in LIHC and KIRC, respectively. The STRIPAK genes serve as oncogenes may due to the methylation heterogeneity. Taken together, our comprehensive molecular analysis of STRIPAK complex provides resource to facilitate the understanding of mechanism and utilize the potential therapies to tumors.

## Introduction

**FIGURE 1:**
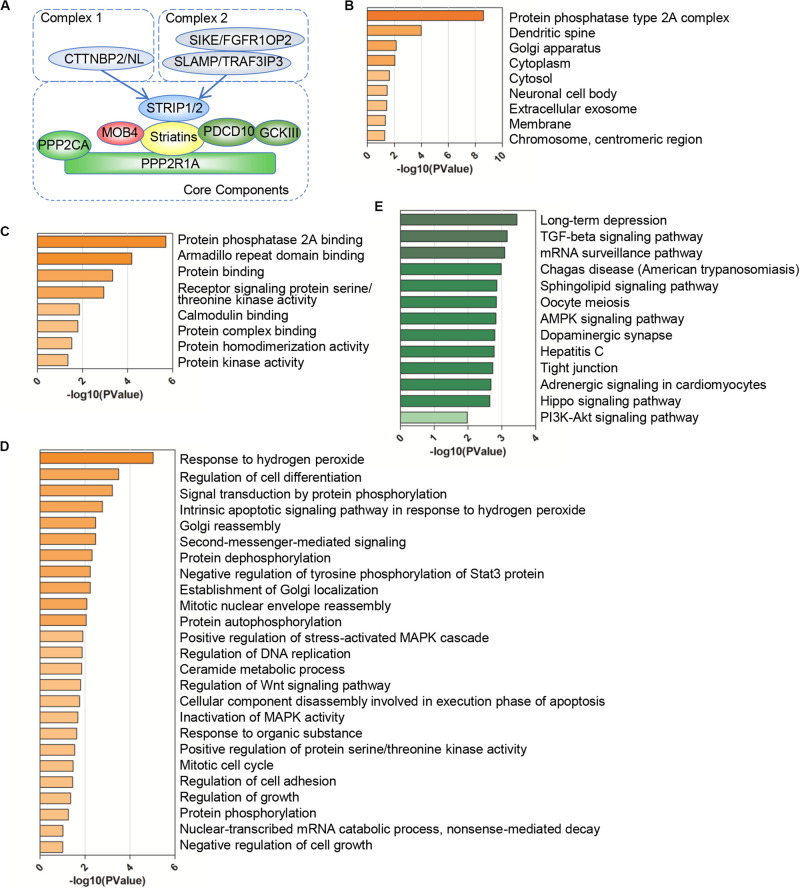
Gene Ontology (GO) and pathway enrichment analysis of the STRIPAK complex. **(A)** Model of the striatin-interacting phosphatase and kinase (STRIPAK) complex. The core components of STRIPAK complex include STRIP1/2, striatins (STRN, STRN3, STRN4), PPP2R1A, PPP2CA, MOB4, PDCD10, and GCKIII (STK24, STK25, STK26). The core complex may interact with CTNNBP2 family proteins to form complex one, or interact with SLMAP and SIKE family to form complex two. **(B–E)** The functional enrichment of 18 STRIPAK genes in cellular components **(B)**, biological process **(C)**, molecular functions **(D)** and KEGG pathway **(E)**, using DAVID online database.

The striatin-interacting phosphatase and kinase (STRIPAK) is a conversed complex during the evolution, which consists of the scaffolding subunit PPP2R1A (also known as PP2AA), catalytic subunit PPP2CA (also known as PP2AC), and regulatory subunit striatins (STRNs) containing STRN, STRN3, and STRN4 (Goudreault et al., 2009; Frost et al., 2012) (Figure 1A and Supplementary Table 1). Striatins recruit striatin-interacting protein 1/2 (STRIP1/2, also known as FAM40A/B), MOB4, PDCD10 (also known as CCM3). PDCD10 interacts with and stabilizes germinal center kinase III (GCK III) family, which consists of three genes, STK24 (also known as MST3), STK25 (also known as YSK1), and STK26 (also known as MST4) (Fidalgo et al., 2010). The investigators predicted that two heteromeric models of the STRIPAK complex, the difference are the binding proteins to STRIP1/2. The interaction to CTTNBP2 family is the complex I, and complex II contains sarcolemmal membrane-associated protein (SLMAP), TRAF3IP3, SIKE1 and FGFR1OP2 (Goudreault et al., 2009). The dynamic assembly of STRIPAK complexes regulates the downstream effectors (Chen et al., 2018; Chen R. et al., 2019; Tang et al., 2019).

The importance of STRIPAK is highlighted in physiological processes (Hwang and Pallas, 2014; Kuck et al., 2019), such as apoptosis (Chen et al., 2009), Golgi assembly (Fidalgo et al., 2010; Kean et al., 2011), and embryo, vascular and neural development (He et al., 2010; Sakuma et al., 2014, 2016; Ultanir et al., 2014; Bazzi et al., 2017; Gil-Ranedo et al., 2019). For example, deletion or mutation of strip1 in mouse embryos caused distinguishable mesoderm morphogenesis (Bazzi et al., 2017), while knockdown of strip in *Drosophila* showed disturbance in early endosome organization (Sakuma et al., 2014). Another regulators in STRIPAK complex, STK24 promotes development of spine synapse via thousand and-one amino acid kinases TAOK1 and TAOK2 associated with Myosin Va axis (Ultanir et al., 2014). The conserved STRIPAK complex regulates the reactivation of *Drosophila* neural stem cells through functioning as a switch to Hippo pathway and InR/PI3K/Akt signaling (Gil-Ranedo et al., 2019). Moreover, STRIPAK plays an important role in human diseases, including heart failure, diabetes, autism, and cerebral cavernous malformation (CCM) (Hwang and Pallas, 2014). For instance, PDCD10 mutations are responsible for CCM (Guclu et al., 2005; Chen et al., 2009), and loss of PDCD10 dissociated ZO-1 and actin via phosphorylation of cortactin, which caused instability of tight junction complex and consequently impairment of brain barrier integrity (Stamatovic et al., 2015). Furthermore, STRIPAK serves as the node of active signaling that contributes to broad implications. The cellular localization of STRIPAK components indicated STRIPAK bridge organelles, such as the nuclear membrane, the centrosome, and Golgi (Guzzo et al., 2004; Fidalgo et al., 2010; Frost et al., 2012; Hwang and Pallas, 2014).

Researchers summarized that STRIPAK complexes play critical roles in cancer (Shi et al., 2016). The components of STRIPAK were identified as the regulators of actomyosin cytoskeleton (Bai et al., 2011; Madsen et al., 2015; Bazzi et al., 2017), determining their roles in the migrate mode of cancer cell via controlling the Ezrin/Radixin/Moesin family proteins (Madsen et al., 2015). Besides the migrate ability, different groups reported that the STRIPAK complex regulates the Hippo pathway in various processes (Ribeiro et al., 2010; Sakuma et al., 2016; Bae et al., 2017; Gil-Ranedo et al., 2019; Tang et al., 2019), including tumor progression (Yu et al., 2015; Chen et al., 2018; Kim et al., 2020). MST1/2 and MAP4Ks, which belong to STE20 kinase family, interact with STRIPAK via core components in *Drosophila* and mammalian cells (Goudreault et al., 2009; Tang et al., 2019). In our previous study, we verified the binding via co-immunoprecipitation assay and founded that STRIPAK initiates the Hippo pathway via RhoA/RHPN1 activation upon upstream signals (Chen R. et al., 2019). Recently, both findings of Madsen lab and our lab showed that STRIPAK complexes controlled oncogenic transformation via regulation of MST1/2 or MAP4K4 (Chen R. et al., 2019; Rodriguez-Cupello et al., 2020).

Since the STRIPAK complex received increased attention in regulation among the signaling network and function in cancers, we provided the analysis of molecular dysregulation and clinical correlation of the STRIPAK complex across cancers using online databases. The results revealed that even though most genes encoding STRIPAK complex did not frequently harbor mutations or copy number alterations across cancers, patients with altered genes showed unfavorable survival. Opposite survival pattern of STRIPAK genes were observed in liver hepatocellular carcinoma and kidney renal clear cell carcinoma. Our study illustrated the role of the STRIPAK complex in cancer, and the discoveries should give the insights of the STRIPAK-related molecular mechanisms and involvement in cancer.

## Materials and Methods

### Gene Set Curation and PPI Network

The genes of STRIPAK complex were curated from the studies using the affinity purification and mass spectrometry approaches (Goudreault et al., 2009). The core STRIPAK genes include PPP2R1A, PPP2CA, STRIP1/2, STRN/3/4, SLMAP, GCKIII family, PDCD10, MOB4, CTTNBP2/NL, FGFR1OP2, TRAF3IP3, and SIKE1. The GCKIII family contains three genes, STK24, STK25, and STK26 (Supplementary Table 1). Protein-protein interaction (PPI) information is evaluated by online Search Tool for the Retrieval of Interacting Genes (STRING version 11.0^1^) (Szklarczyk et al., 2015). Date of the query was 2020-06-03. The organism was “*Homo sapiens*,” minimum required interaction score was 0.400 (medium confidence) and without clustering. The results of network statistics were from the “Analysis.” The visualization and analysis of PPI network is performed by Cytoscape (version 3.8.0).

### GO and KEGG Enrichment Analyses of STRIPAK Genes

The Database for Annotation, Visualization and Integrated Discovery (DAVID^2^) (Dennis et al., 2003) (version 6.8) was applied for the functional and pathway enrichment of proteins encoded by STRIPAK genes. Gene Ontology (GO) analysis is a common bioinformatics tool for large-scale analysis of functional enrichment. The classifications of gene functions contain biological process (BP), molecular function (MF), and cellular component (CC). GO annotations of STRIPAK genes were performed using a DAVID online tool. KEGG (Kyoto Encyclopedia of Genes and Genomes) is a database resource, which provides large-scale information in molecular level and facilitates scientists to manipulate high-level functions and biological processes (Kanehisa and Goto, 2000). KEGG pathway analysis of STRIPAK genes was performed using the KOBAS online analysis database^3^ (Xie et al., 2011) (version 3.0), and date of the query was 2019-11-02. *p*-value of <0.05 was considered statistically significant.

### Somatic Copy-Number Alteration (SCNA) and Somatic Mutation Analysis

The SCNA and somatic mutation of STRIPAK genes were analyzed and depicted using cBioPortal^4^ (Gao et al., 2013). All of analysis was performed on 18 STRIPAK genes in the set of 32 non-redundant TCGA PanCancer Atlas studies containing 10967 samples. The date of the query was 2020-06-10.

### Prognostic Power of STRIPAK Genes

The overall survival (OS) and disease-free survival (DFS) of STRIPAK genes were analyzed by cBioPortal (Gao et al., 2013), GEPIA2^5^ (Tang et al., 2017), Kaplan–Meier plotter^6^, UALCAN^7^ (Chandrashekar et al., 2017), LOGpc^8^ (Xie et al., 2019) and MethSurv^9^ (Modhukur et al., 2018) which are the online tools for visualization, evaluation and download of large-scale cancer-related genomics data sets. The Kaplan-Meier curves in cBioPortal were applied to evaluate the prognostic power of STRIPAK genes with genetic alterations in patients harboring cancer. The survival map was generated by GEPIA2 to screen the prognostic value of STRIPAK genes in cancers, which we selected “0.05” as the significance level, and “Median” as the group cutoff. The survival map of GSE29609 and GSE76427 was generated by R packages pheatmap. The prognostic value of STRIPAK genes in 371 samples of liver cancer and 530 samples of kidney renal clear cell carcinoma (KIRC) were performed using from the Kaplan–Meier plotter mRNA (RNAseq) pan-cancer database. The expression level of each gene was grouped by best cutoff and months of survival were selected as survival time units. *p*-value of < 0.05 was considered statistically significant. The combined survival analysis of TCGA and GEO database were performed using the online consensus survival for LIHC (OSlihc) and KIRC (OSkirc) of LOGpc, which data source was “combined” and split patients by “Upper 50%” or “Upper 25%.” The survival analysis of DNA methylation was performed using MethSurv, which split by “best” option to dichotomize methylation profiles without adjust for co-variates.

### Expression of STRIPAK Genes in Human Samples and Cancer Cell Lines

The expression profiles of STRIPAK genes in normal and tumor tissues between LIHC and KIRC were analyzed and/or showed using Oncomine^10^ (Rhodes et al., 2004), GEPIA2, UALCAN, the Cancer Proteome Altas (TCPA^11^) (Li et al., 2013) and R packages pheatmap. We used the following parameters of Multiple Genes Comparison in GEPIA2: “Yes” for Log Scale, and “Matched TCGA normal data.” To identify the differentially expression proteins between LIHC and KIRC, we took advantages of the function named “Differential Analysis” under “Individual Cancer Analysis” in TCPA. Oncomine is an online cancer microarray database that provides a platform to discover genome-wide expression differences. We screened tumor studies in Oncomine database with include criteria as follows: (a) Cancer Type: LIHC or KIRC, (b) “Gene: PPP2R1A,” (c) “Analysis Type: Cancer vs. Normal Analysis” and (d) Threshold setting condition (*p* < 0.05, Fold change >1.5, gene rank = all). The correlation between the clinical-pathological parameters and expression level or methylation profiles of STRIPAK were analyzed using UALCAN. The co-expression of TRAF3IP3 and Src family tyrosine kinase (LCK) were analyzed using cBioportal and TCPA. The DNA methylation profiles of TRAF3IP3 in KIRC were performed using the function named “Gene visualization” in MethSurv.

The comparison of STRIPAK genes between liver cancer cell lines and kidney cancer cell lines was performed based on the data extracted from the Cancer Cell Line Encyclopedia (CCLE^12^) (Barretina et al., 2012). We extracted RNAseq data of liver and kidney cancer cell lines from CCLE with the criteria as follows, (a) matched cancer type; (b) none problematic or contaminated; (c) had values. The annotations of cell lines were performed according to the Expasy Cellosaurus^13^. Finally, 21 kinds of liver cancer cell lines and 12 kinds of kidney cancer cell lines were included in our study.

### Statistical Analysis

The statistical tests were performed using R software (version 3.6.1). We used *t-test* (two-tailed) or *Mann-Whitney* test for comparison between two groups. We used the *Schoenfeld* individual test to evaluate the proportional hazards assumption for the fit of Cox regression model. The survival analysis of survival was performed using univariate and multivariate Cox proportional hazards model, which split by optimal cutoff values, which were generated by the “surv_cutpoint” function of R package survminer (Kassambara et al., 2019), to dichotomize expression level. A value of *p* < 0.05 was considered statistically significant, ^∗^*p* < 0.05, ^∗∗^*p* < 0.01, ^∗∗∗^*p* < 0.001.

## Results

### Gene Oncology and Pathway Enrichment of STRIPAK

To verify our data set (Figure 1A), we constructed the PPI network of STRIPAK complex via STRING online tool. The average local clustering coefficient of the PPI network is 0.992 and *p*-value of PPI enrichment is less than 1.0e-16, which suggests that the connections of STRIPAK proteins are dense. To understand deeply among STRIPAK complex, we performed GO functions and KEGG pathway enrichment analysis using online tools of DAVID and KOBAS. There was no surprise that the enrichment showed that STRIPAK relates to the protein phosphatase type 2A complex and regulates the signal transduction by phosphorylation and dephosphorylation (Figures 1B–D).

Gene ontology enrichment analysis showed that genes encoded STRIPAK were enriched in cellular components, including dendritic spine and Golgi apparatus (Figure 1B); biological processes, including armadillo repeat domain binding and receptor signaling protein serine/threonine kinase activity (Figure 1C); and molecular functions, including response to hydrogen peroxide and regulation of cell differentiation (Figure 1D). KEGG pathway analysis showed that STRIPAK genes were significantly enriched in long-term depression, TGF-beta signaling pathway and mRNA surveillance pathway (Figure 1E).

### Molecular Alteration Landscape of the STRIPAK Complex

**FIGURE 2:**
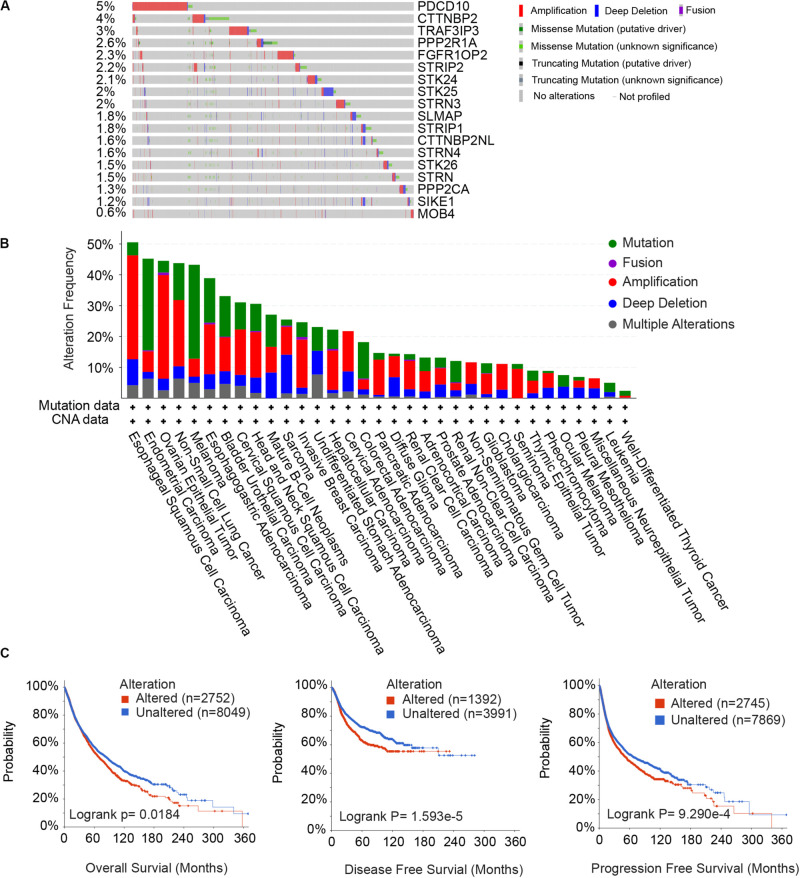
Somatic alterations of the STRIPAK complex. **(A,B)** Waterfall plots **(A)** and distribution **(B)** of gene mutation and copy number alteration of 18 STRIPAK genes. **(C)** Kaplan-Meier analysis of overall survival, disease-free survival and progression-free survival for patients with or without genetic mutations of STRIPAK. All data are from cBioportal database.

Since the STRIPAK complex is important for physiological and pathological processes, we focused on the genetic alterations of STRIPAK genes across cancers. We calculated the somatic copy number alteration (SCNA) and mutation frequency in pan-cancer cohort of 10967 patients using cBioportal (Figures 2A,B). The overall DNA aberration was low, ranging from 0.6 to 5%. PDCD10 ranked the highest altered frequency, followed by CTTNBP2, TRAF3IP3, and PPP2R1A. Amplification was the predominant type of STRIPAK aberration. Surprisingly, altered STRIPAK genes are present in 50.53% (48/95 cases) of patients with esophageal squamous cell carcinoma and 38.91% (200/514 cases) of patients with esophagogastric adenocarcinoma (Figure 2B). Thus, STRIPAK genes with DNA aberration might be involved in esophageal carcinoma. The aberration of STRIPAK genes showed the unfavorable overall survival (OS), disease-free survival (DFS) and progression-free survival (Figure 2C), which suggested the significance of STRIPAK dysregulation across cancers on the account for the landscape of somatic alteration and mutation.

### Prognostic Power and Differential Expression of STRIPAK Genes Across Cancers

**FIGURE 3:**
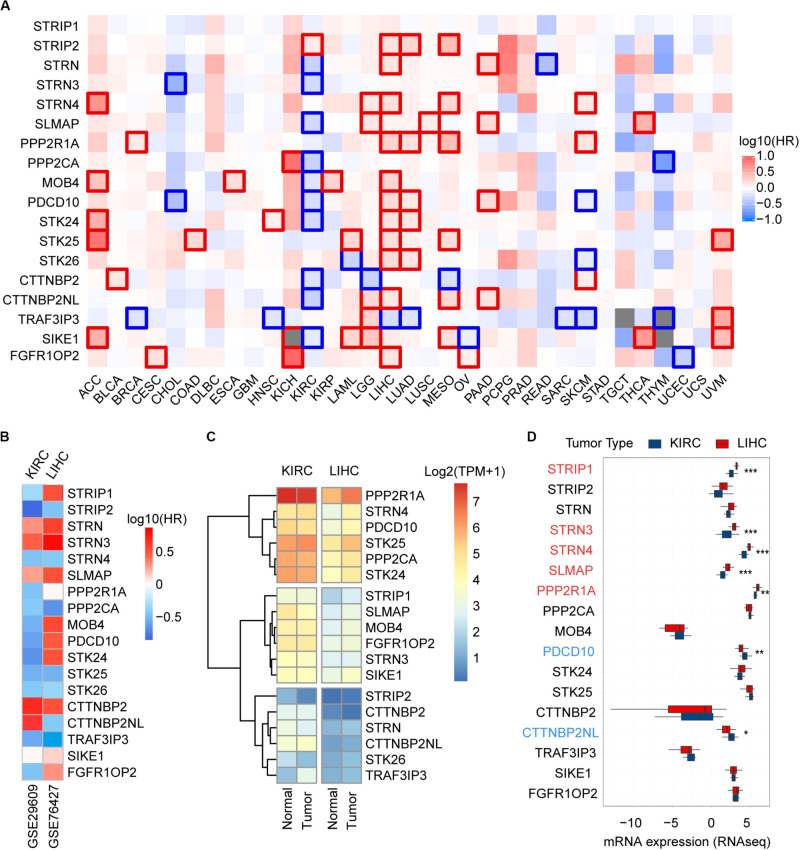
Prognostic power and expression level of the STRIPAK genes in cancers. **(A)** Kaplan-Meier analysis of overall survival according to the expressions of STRIPAK genes in TCGA data using GEPIA online tool without false discovery rate (FDR) adjustment. The boxes with framed indicated significant results (*p* < 0.05). **(B)** Survival analysis of STRIPAK genes using data of GSE29609 and GSE76427. **(C)** Expression level of STRIPAK genes in normal and tumor tissues of KIRC and LIHC. Log2(TPM+1) of TCGA data were showed. TPM is short for transcript per million. **(D)** Differences in mRNA expression between cell lines of LIHC and KIRC according to the CCLE database. Genes in color depicts statistical significance, genes in red depicts the higher expression level of liver cancer cell lines, and genes in blue depicts that kidney cancer cell lines expressed higher. *p < 0.05, **p < 0.01, ***p < 0.001.

To unravel prognostic value of STRIPAK genes across cancer types, we screened the correlation of their expression with patients’ survival using GEPIA2 and Kaplan–Meier plotter. The abbreviations of cancer type were summarized in Supplementary Table 2. The results of survival analysis showed that many STRIPAK genes had significant survival across cancer (Figure 3A and Supplementary Figures 1–3). Liver hepatocellular carcinoma (LIHC) had 16 significant genes, which ranked the highest cancer type, followed by kidney renal clear cell carcinoma (KIRC) that had 15 significant genes (Supplementary Table 3 and Supplementary Figures 2, 3). Even though only 14.48% (151/1043) cases harbored the genetic alterations, LIHC patients with DNA aberration showed unfavorable overall survival and disease-free survival (Supplementary Figure 2D).

Surprisingly, opposite patterns were observed in the above top 2 cancer types with or without false discovery rate (FDR) adjustment of *p*-value (Figure 3A and Supplementary Figures 1–3). Opposite to LIHC patients, KIRC patients with high expressions of STRIPAK genes showed favorable survival (Figure 3A and Supplementary Figure 3). To confirm the survival prediction patterns of STRIPAK between KIRC and LIHC, we analyzed GSE29609 and GSE76427 by splitting the group by optimum value of gene expression level. The result was consistent with survival map generated by GEPIA2 (Figure 3B and Supplementary Figures 2C, 3C). To conclude, the high expression level of whole STRIPAK complex might serve as the tumor suppressor in KIRC, while serve as oncogenic role in LIHC.

To confirm whether this pattern was due to the expression difference exists in two cancer types, we compared the mRNA expression level with the normal and tumor tissues using the same database. The results showed that STRIPAK expressed higher in KIRC tissues than in LIHC tissues, but seldom genes were found as significant difference between normal and tumor tissues in each cancer type (Figures 3C, 5A, 6A). The differentially expression was also detected between cancer cell lines between KIRC and LIHC (Figure 3D). Thus, these results indicated that STRIPAK complex might relate to prognosis of patients with cancer, and play opposite roles between KIRC and LIHC.

### Identify Potential Targets to Explore the Role of STRIPAK Complex Between KIRC and LIHC

**FIGURE 4:**
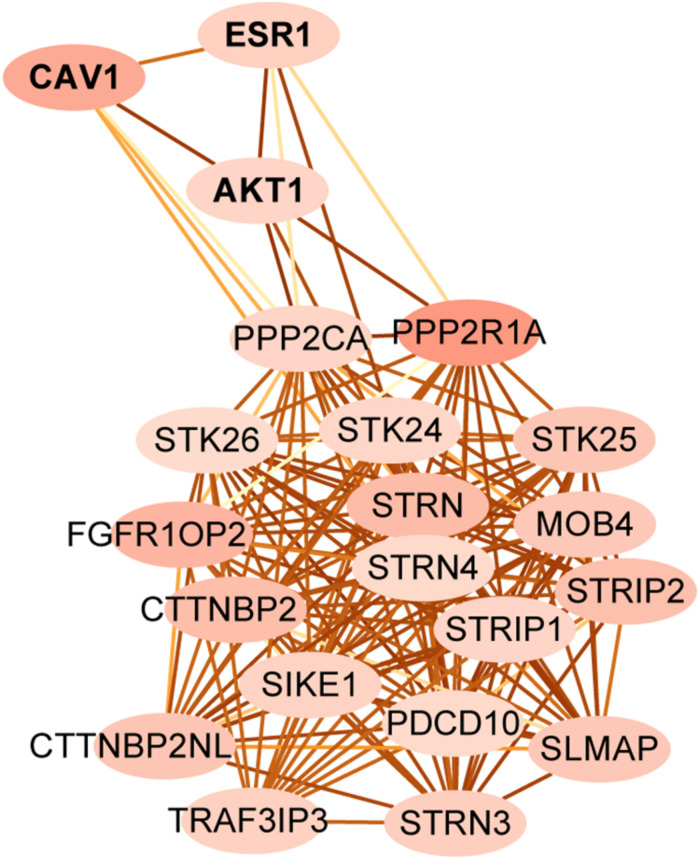
The protein-protein interaction subnetwork between STRIPAK genes and different expression level of proteins between LIHC and KIRC. Map node color to indegree, and low values to brighter colors. Map edge color to the combined_score, which means edges with lighter color have fewer protein-protein associations.

Striatin-interacting phosphatase and kinase is a multifunctional complex, which is regulated by many upstream signals and regulates many downstream pathways (Hwang and Pallas, 2014). To identify targets that associate with STRIPAK in KIRC and LIHC, we first compared the expressed proteins between two cancer types using TCPA. Two hundred and seventeen entries were reported as the differentially expressed protein. Since the total protein and phosphorylated protein were encoded as same gene, we submitted 174 genes of 217 entries and 18 STRIPAK genes to STRING for analyzing the PPI network. (Data not shown.) Then, we selected PPP2R1A as the central node to create subnetwork (Figure 4) because of the value of indegree. The connections of STRIPAK components were dense according to the subnetwork. AKT serine/threonine kinase 1 (AKT1), caveolin1 (CAV1) and estrogen receptor 1 (ESR1) were in the subnetwork, and the expression level of above proteins expressed higher in LIHC than KIRC (Table 1). The results indicated that AKT1, CAV1 and ESR1 could be the targets to explore the function of STRIPAK between KIRC and LIHC.

**TABLE 1:** Different expression levels of selected protein between KIRC and LIHC using TCPA.

**Protein_Marker_ID**	**Gene**	RPPA protein abundances*		***p*-value**
		**KIRC**	**LIHC**	
AKT	AKT1,AKT2,AKT3	−0.71841	−0.042755	5.25E-58
AKT_pT308	AKT1,AKT2,AKT3	−0.36531	0.24294	2.59E-38
AKT_pS473	AKT1,AKT2,AKT3	−0.02963	0.51806	5.31E-19
CAVEOLIN1	CAV1	−0.28233	1.215	5.81E-80
ERALPHA	ESR1	−0.72737	−1.6739	5.13E-63
ERALPHA_pS118	ESR1	−0.32523	−0.44399	2.56E-09

*RPPA: Reverse phase protein array

### PPP2R1A Might Serve as the Oncogene in LIHC

**FIGURE 5:**
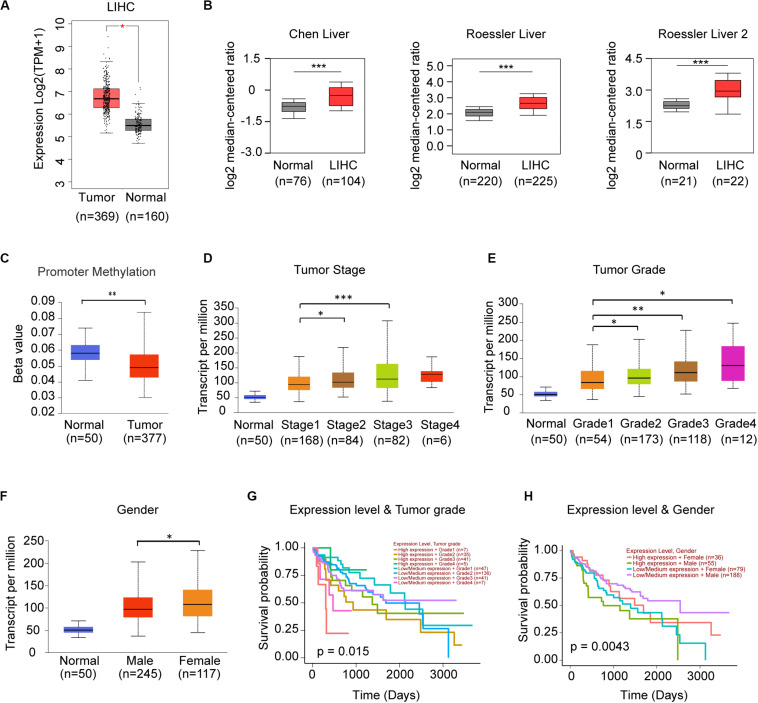
PPP2R1A might serve as the oncogene in LIHC. **(A)** Comparison of the mRNA expression level of PPP2R1A between normal and tumor tissues in TCGA-LHC database using GEPIA. **(B)** The mRNA expression level of PPP2R1A in the studies of LIHC in Oncomine database. **(C–F)** Expression of PPP2R1A in LIHC based on promoter methylation level **(C)**, individual cancer stages **(D)**, tumor grade **(E)**, and gender **(F)**. The Beta value indicates level of DNA methylation, and 0 means unmethylated, and 1 means fully methylated. **(G,H)** Kaplan-Meier plot showing the significant difference of the effect between PPP2R1A expression level, and tumor grade **(G)** and gender **(H)** in LIHC patients, respectively. *p < 0.05, **p < 0.01, ***p < 0.001.

Since frequency of DNA alteration was low in each genes of STRIPAK complex in LIHC (Data not shown), we speculated the gene expression might be responsible for the prognostic power. We compared the expression level of STRIPAK genes between normal tissue and tumor tissue via GEPIA2, discovered that only PPP2R1A were observed significant difference (Figure 5A). In Oncomine database, we identified 3 of 8 studies that showed significant increased expression in tumor tissue, including Chen liver (GSE3500) and Roessler liver (GSE14520) (Figure 5B). The promoter methylation level of PPP2R1A was not parallel to the expression level, lower methylation were observed in LIHC tumor tissues while the expression level is higher (Figures 5A,C). Expression of PPP2R1A significantly correlated with tumor stage, tumor grade as well as gender (Figures 5D–F). Then, we evaluated the prognostic role of expression level of PPP2R1A and clinical parameters in LIHC. The results showed that PPP2R1A expression level accompanied with tumor grade or gender significantly correlated with overall survival (Figures 5G,H). Taken together, PPP2R1A might serve as an oncogene in LIHC.

### TRAF3IP3 Might Play an Oncogenic Role in KIRC

**FIGURE 6:**
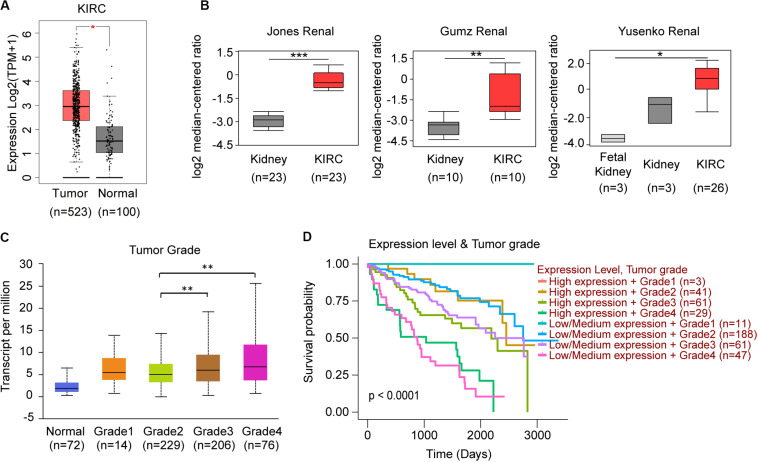
TRAF3IP3 might play an oncogenic role in KIRC. **(A)** Comparison of KIRC normal and tumor tissues of TRAF3IP3 in TCGA database using GEPIA. **(B)** TRAF3IP3 expression in the studies of KIRC in Oncomine database. **(C,D)** Expression and Kaplan-Meier plot of TRAF3IP3 in KIRC based on tumor grade using UALCAN. *p < 0.05, **p < 0.01, ***p < 0.001.

When we compared the expression between normal kidney and KIRC tissues, only TRAF3IP3 showed significant difference (Figure 6A). In the analysis of 6 studies containing KIRC in Oncomine database, 3 showed significant increased expression of TRAF3IP3. (Figure 6B) The 3 studies were Jones Renal (GSE15641), Gumz Renal (GSE6344), and Yusenko Renal (GSE11151). Except tumor grade (Figure 6C), the correlation between the expression of TRAF3IP3 and clinicopathological features showed no significance performed by UALCAN (Data not show). KIRC patients with low or medium expression of TRAF3IP3 and low tumor grade possibly had a good overall survival (Figure 6D).

**FIGURE 7:**
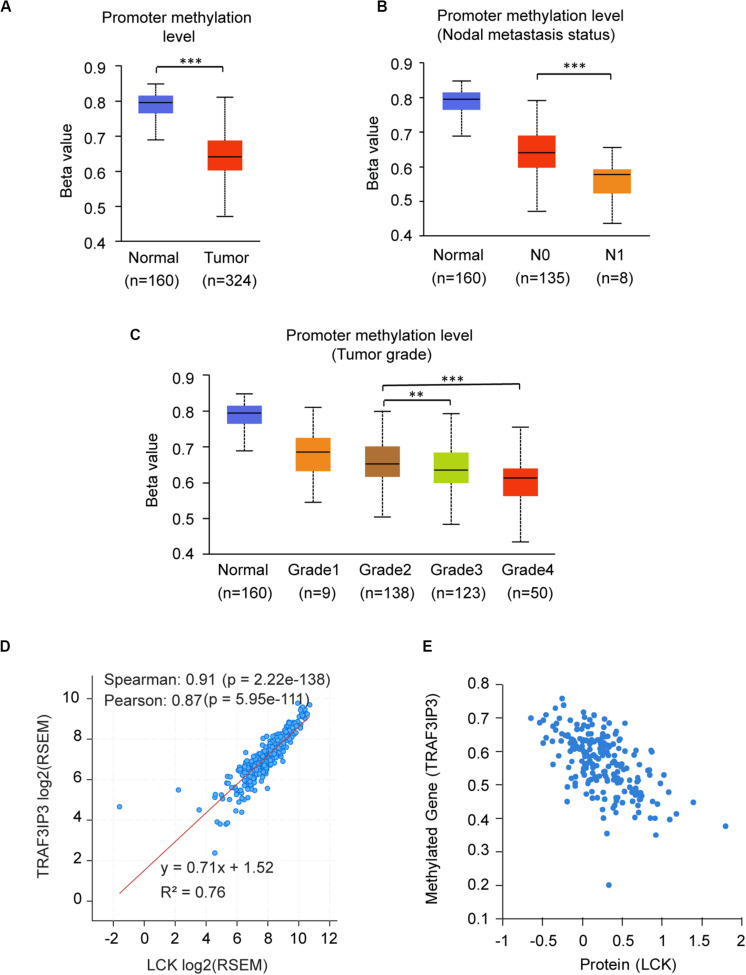
The promoter methylation level of TRAF3IP3 is a potential regulator in KIRC. **(A–C)** The promoter methylation level of TRAF3IP3 in KIRC based on sample types **(A)**, nodal metastasis status **(B)** and tumor grade **(C)** using UALCAN. **(D)** The correlation of mRNA expression level between LCK and TRAF3IP3 using cBioportal. **(E)** The protein level of LCK was predicted to related to the methylation level of TRAF3IP3 in KIRC using TCPA. The Beta value indicates level of DNA methylation, and 0 means unmethylated, and 1 means fully methylated. The value of RNA-Seq by Expectation Maximization (RSEM) is provided by cBioportal. **p < 0.01, ***p < 0.001.

**FIGURE 8:**
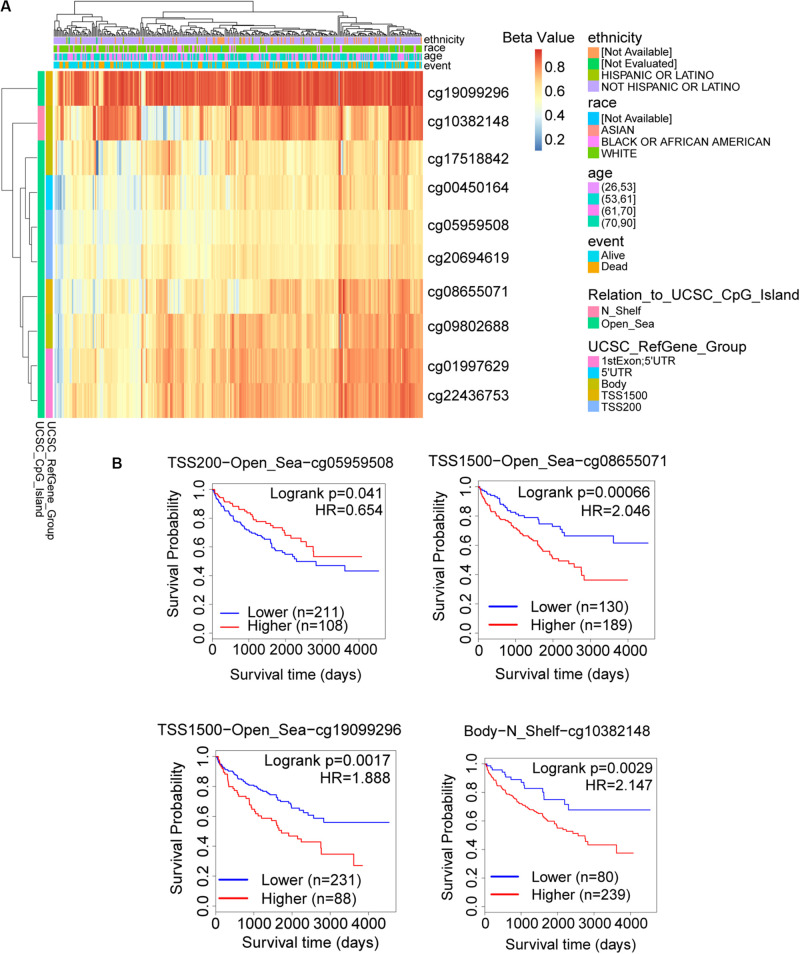
The possible functional site of DNA methylation in TRAF3IP3. **(A)** The heatmap for methylated TRAF3IP3 in TCGA-KIRC using MethSurv. **(B)** Kaplan meier plots of single CpG site that had significant prognostic value for TRAF3IP3 in TCGA-KIRC. The Beta value indicates level of DNA methylation, and 0 means unmethylated, and 1 means fully methylated.

The promoter methylation of TRAF3IP3 was observed higher in normal tissue than KIRC tissue (Figure 7A), and related with tumor grade and nodal metastasis status (Figures 7B,C). The results suggested that DNA methylation of TRAF3IP3 involved in the tumorgenesis of KIRC. To better know the driver genes of TRAF3IP3, we searched the TCPA database and found the protein level of Src family tyrosine kinase (LCK) negatively correlated with the methylated TRAF3IP3 (*R* = −0.57479, *p* < 0.05), while LCK positively correlated with TRAF3IP3 in mRNA expression level (Figures 7D,E). Then, we identified single CpG sites (cg05959508, cg08655071, cg19099296, and cg10382148) as potential functional sites of TRAF3IP3 through MethSurv because of the significant prognostic value (Figure 8). Taken together, the methylation of TRAF3IP3 might be a candidate to play a role in tumorigenesis of KIRC.

## Discussion

Cancer ranks as top of the leading causes of death around world in the 21st century, and the rapidly increasing incidence and mortality of cancer urge people to reduce burden through prevention and intervention against tumor (Bray et al., 2018). As the emerging star in biological process, limited articles reported STRIPAK complex play a role in tumorigenesis recently. Thus we performed molecular profiles of STRIPAK complex across cancer using online tools to analyze multi-dimensional characterization of clinical data. Unlike previous researches (Madsen et al., 2015; Chen et al., 2018; Chen R. et al., 2019), which focused on few cancer types, our results revealed that the DNA aberrations, expression and methylation, and prognostic power of STRIPAK complex across over 20 cancer types. Furthermore, PPP2R1A and TRAF3IP3 were identified as the novel potential oncogenic genes through the integrated analysis in liver hepatocellular carcinoma (LIHC) and kidney renal clear cell carcinoma (KIRC), respectively.

One significant contribution of this study is that we illustrated dysregulated STRIPAK complex drove diverse mechanisms to involve in the tumor progression. First is the mutation-driven mechanism. Our analysis revealed that poor survivals were showed in patients harboring altered STRIPAK genes and high-frequency aberrations were observed in specific cancer types, such as esophageal carcinoma. The individual mutated STRIPAK gene expressed tumor-related phenotype (Madsen et al., 2015; Kim et al., 2020). Second is the protein-protein regulation. In this study, we identified AKT1, CAV1 and ESR1 as candidates to discover the function of STRIPAK complex between KIRC and LIHC. It is well established that the ATK1 is one of major substrates for protein phosphatase 2A (PP2A) (Seshacharyulu et al., 2013), which is indicated as the function of STRIPAK via the GO enrichment analysis (Figure 1). Third is post-transcriptional regulation. In this study, few differences of expression level were found in STRIPAK genes across cancer. Hence, we turned to assess protein post-transcriptional modifications. There were significantly comparable methylation levels of PPP2R1A and TRAF3IP3 between normal and tumor tissue. Other modifications could be studied in the future to dig up the mechanisms of STRIPAK complex. The last is dynamic complex assembly. Even though the core components of STRIPAK complex were well known, the interaction among components were still elucidated. Dynamic assembly of STRIPAK regulates the cell proliferation, migration and invasion of cancer (Chen et al., 2018; Chen R. et al., 2019; Tang et al., 2019; Rodriguez-Cupello et al., 2020). This could explain why the expression homogeneity was found between the normal and tumor samples.

DNA methylation is an impactful epigenetic modification in STRIPAK complex. Since the contrary phenotypes of PPP2R1A and TRAF3IP3 were observed in expression level and methylation level (Figures 5A,C, 6A, 7A), there is a reasonable assumption that PPP2R1A or TRAF3IP3 losses methylation may induce its stabilization. The methylation level of TRAF3IP3 gradually reduced with increased tumor grade and lymph node metastasis status (Figures 7B,C). Thus, we proposed that the methylation of STRIPAK genes might drive tumor progression via the downstream effectors as well as methylated YAP promotes tumorigenesis via increasing YAP/TEAD transcription (Fang et al., 2018). We also proposed that Src family tyrosine kinase (LCK) reversely regulated TRAF3IP3 methylation based on correlation analysis using cBioportal and TCPA (Gao et al., 2013; Chen M. M. et al., 2019) (Figures 7D,E). LCK, which belongs to Src-like non-receptor tyrosine kinase family, might lay upstream of TRAF3IP3, because knockout Traf3ip3 in CD4^+^CD8^+^ double-positive thymocytes did not show difference in phosphorylation and expression of LCK compared with Traf3ip3^+/+^ cells (Zou et al., 2015). Moreover, the reversibility of DNA methylation is the promising targets for therapy (Kulis and Esteller, 2010).

There were many shortcomings in this study. First, we did not apply the experimental approaches to represent our computational results. However, the results from different groups could illustrate our discoveries. For example, our analysis uncovered that cancer patients with altered STRIPAK genes showed unfavorable survival (Figure 2C). Other group reported that the mutants of truncated STRIP2, which were found in lung adenocarcinoma and uterine carcinoma, loss the ability to bind with PPP2CA and drove cell contraction, underlying their role in cancer (Madsen et al., 2015). The MST4-MOB4 complex disassociates the assembly of MST1-MOB1 complex in pancreatic cancer, which promote cell proliferation and migration via inhibition of LATS and YAP phosphorylation (Chen et al., 2018). In addition, more information is needed to explain confused results. On the contrary of the TCGA data, 71.43% (5/7 significant genes) showed higher expression level in KIRC cell lines than LIHC cell lines, including STRIP1, STRN3, STRN4, SLMAP, and PPP2R1A (Figures 3C,D). The comparison suggested that we should be more careful for verification and implication of STRIPAK complex on cell lines based on expression-related phenotypes.

To conclude, the dysregulation of STRIPAK complex play crucial roles in different cancer contexts, especially in liver hepatocellular carcinoma and kidney renal clear cell carcinoma. The valuable resource derived from our systematic and molecular analyses of this complex will advance mechanism comprehension in tumorigenesis and potential therapies to cancers.

## Data Availability Statement

The datasets generated for this study can be found in the TCGA (https://cancergenome.nih.gov/), CCLE (https://portals.broadinstitute.org/ccle/), Oncomine (http://www.oncomine.com/), and TCPA (https://tcpaportal.org/tcpa/index.html).

## Author Contributions

RX designed the experiments, analyzed data, and wrote and revised the manuscript. FW collected and analyzed data. YQ supervised the project. All authors discussed the results and commented on the manuscript.

## Conflict of Interest

The authors declare that the research was conducted in the absence of any commercial or financial relationships that could be construed as a potential conflict of interest.

## Footnotes

1 https://string-db.org/

2 http://david.ncifcrf.gov

3 http://kobas.cbi.pku.edu.cn/

4 http://www.cbioportal.org/

5 http://gepia2.cancer-pku.cn/

6 http://kmplot.com/analysis/

7 http://ualcan.path.uab.edu/

8 http://bioinfo.henu.edu.cn/DatabaseList.jsp

9 https://biit.cs.ut.ee/methsurv/

10 http://www.oncomine.com/

11 https://tcpaportal.org/tcpa/index.html

12 https://portals.broadinstitute.org/ccle/

13 https://web.expasy.org/cellosaurus/

## Supplementary Material

FIGURE S1 Prognostic Power of the STRIPAK genes in cancers using GEPIA2. Kaplan-Meier analysis of overall survival with false discovery rate (FDR) adjustment **(A)** and disease-free survival with **(B)** or without FDR adjustment **(C)** according to the expressions of STRIPAK genes in TCGA data using GEPIA2 online tool. The boxes with framed were significant results (*p* < 0.05).

FIGURE S2 Kaplan-Meier survival plots of STRIPAK genes in liver hepatocellular carcinoma (LIHC). **(A,B)** Kaplan-Meier plot showing the significant difference of STRIPAK genes in unfavorable **(A)** and favorable **(B)** survivial of patients with LIHC. **(C)** The survival analysis of overall survival for LIHC patients in combined studies of TCGA and GEO databases. **(D)** Kaplan-Meier analysis of overall survival and disease-free survival for LIHC patients with or without genetic alterations of STRIPAK genes in 7 non-redundant studies via cBioPortal.

FIGURE S3 Kaplan-Meier survival plots of STRIPAK genes in kidney renal clear cell carcinoma (KIRC). **(A,B)** Kaplan-Meier plot showing the significant difference of STRIPAK genes in unfavorable **(A)** and favorable **(B)** survivial of patients with KIRC. **(C)** The survival analysis of overall survival for KIRC patients in combined studies of TCGA and GEO databases.

TABLE S1 STRIPAK components in *Homo sapiens*.

TABLE S2 The abbreviations of cancers in this study.

TABLE S3 The prognostic information of the 18 genes of the STRIPAK complex in 21 types of cancer in Kaplan-Meier plotter.
