# Supplementary figures and images for: The Dysregulation and Prognostic Analysis of STRIPAK Complex Across Cancers
Source: Front Cell Dev Biol. 2020 Jul 10;8:625. doi: 10.3389/fcell.2020.00625 (PMC7365848; doi:10.3389/fcell.2020.00625)

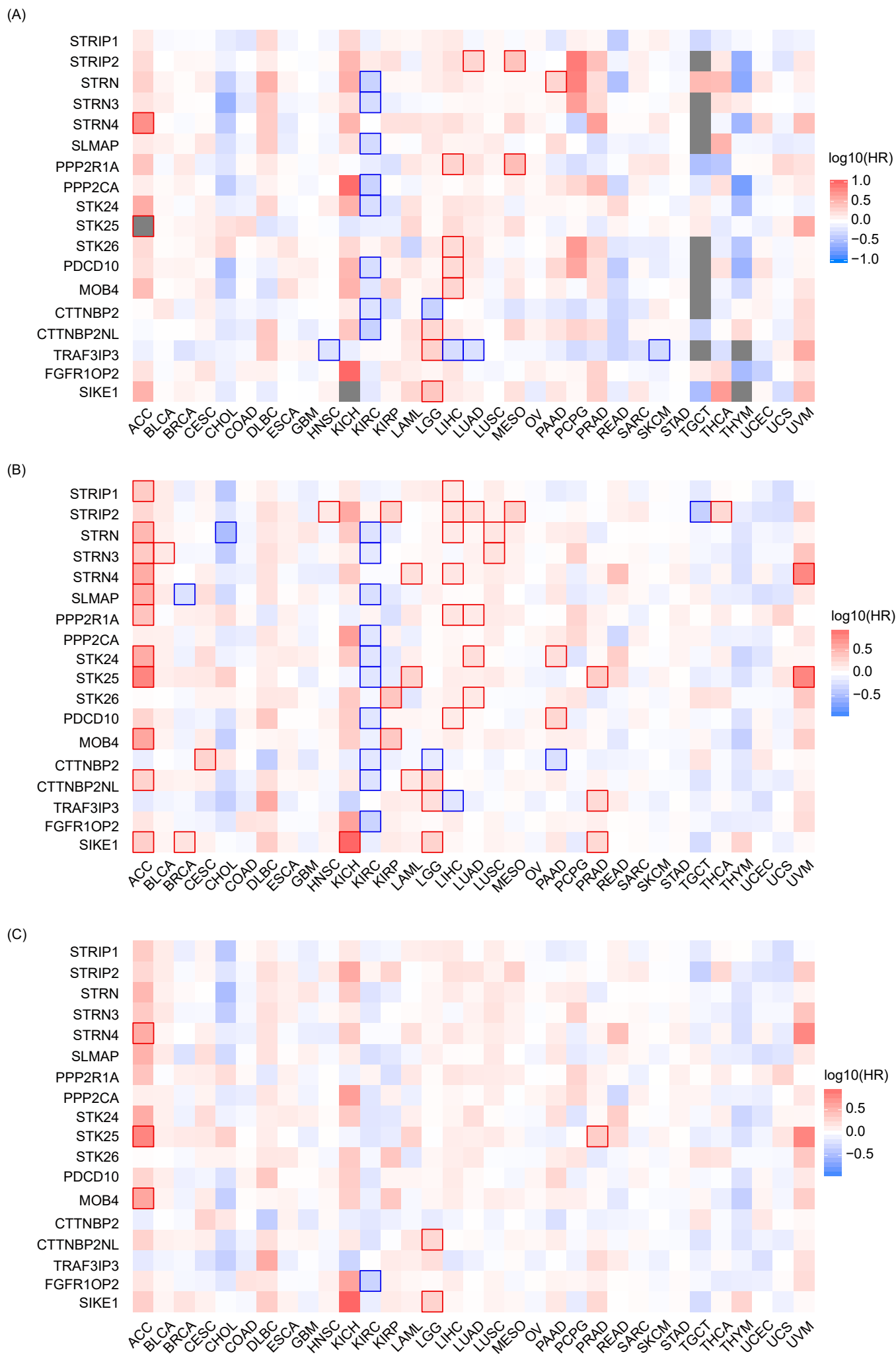

Supplementary Figure 1. Prognostic power of STRIPAK genes using GEPIA2.

Supplement: FIGURE S1 — Prognostic Power of the STRIPAK genes in cancers using GEPIA2. Kaplan-Meier analysis of overall survival with false discovery rate (FDR) adjustment (A) and disease-free survival with (B) or without FDR adjustment (C) according to the expressions of STRIPAK genes in TCGA data using GEPIA2 online tool. The boxes with framed were significant results (p < 0.05). [file Image_1.pdf]
