# Supplementary material for: The Dysregulation and Prognostic Analysis of STRIPAK Complex Across Cancers
Source: Front Cell Dev Biol. 2020 Jul 10;8:625. doi: 10.3389/fcell.2020.00625 (PMC7365848; doi:10.3389/fcell.2020.00625)
Supplement: FIGURE S2 — Kaplan-Meier survival plots of STRIPAK genes in liver hepatocellular carcinoma (LIHC). (A,B) Kaplan-Meier plot showing the significant difference of STRIPAK genes in unfavorable (A) and favorable (B) survivial of patients with LIHC. (C) The survival analysis of overall survival for LIHC patients in combined studies of TCGA and GEO databases. (D) Kaplan-Meier analysis of overall survival and disease-free survival for LIHC patients with or without genetic alterations of STRIPAK genes in 7 non-redundant studies via cBioPortal. [file Image_2.pdf]

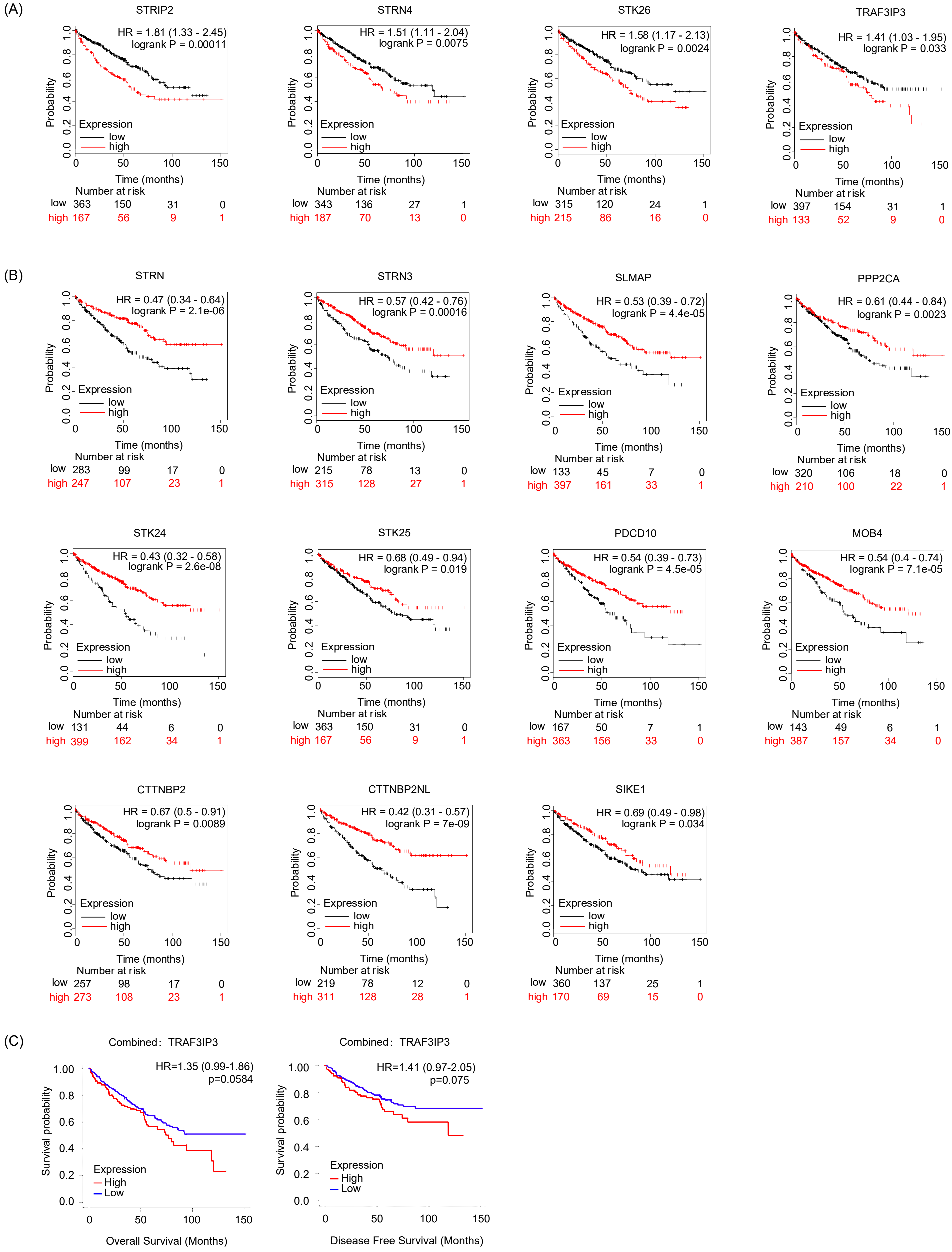

Supplementary Figure 3. Kaplan-Meier survival plots of STRIPAK genes in kidney renal clear cell carcinoma(KIRC).
