# Supplementary material for: The Dysregulation and Prognostic Analysis of STRIPAK Complex Across Cancers
Source: Front Cell Dev Biol. 2020 Jul 10;8:625. doi: 10.3389/fcell.2020.00625 (PMC7365848; doi:10.3389/fcell.2020.00625)
Supplement: FIGURE S3 — Kaplan-Meier survival plots of STRIPAK genes in kidney renal clear cell carcinoma (KIRC). (A,B) Kaplan-Meier plot showing the significant difference of STRIPAK genes in unfavorable (A) and favorable (B) survivial of patients with KIRC. (C) The survival analysis of overall survival for KIRC patients in combined studies of TCGA and GEO databases. [file Image_3.pdf]

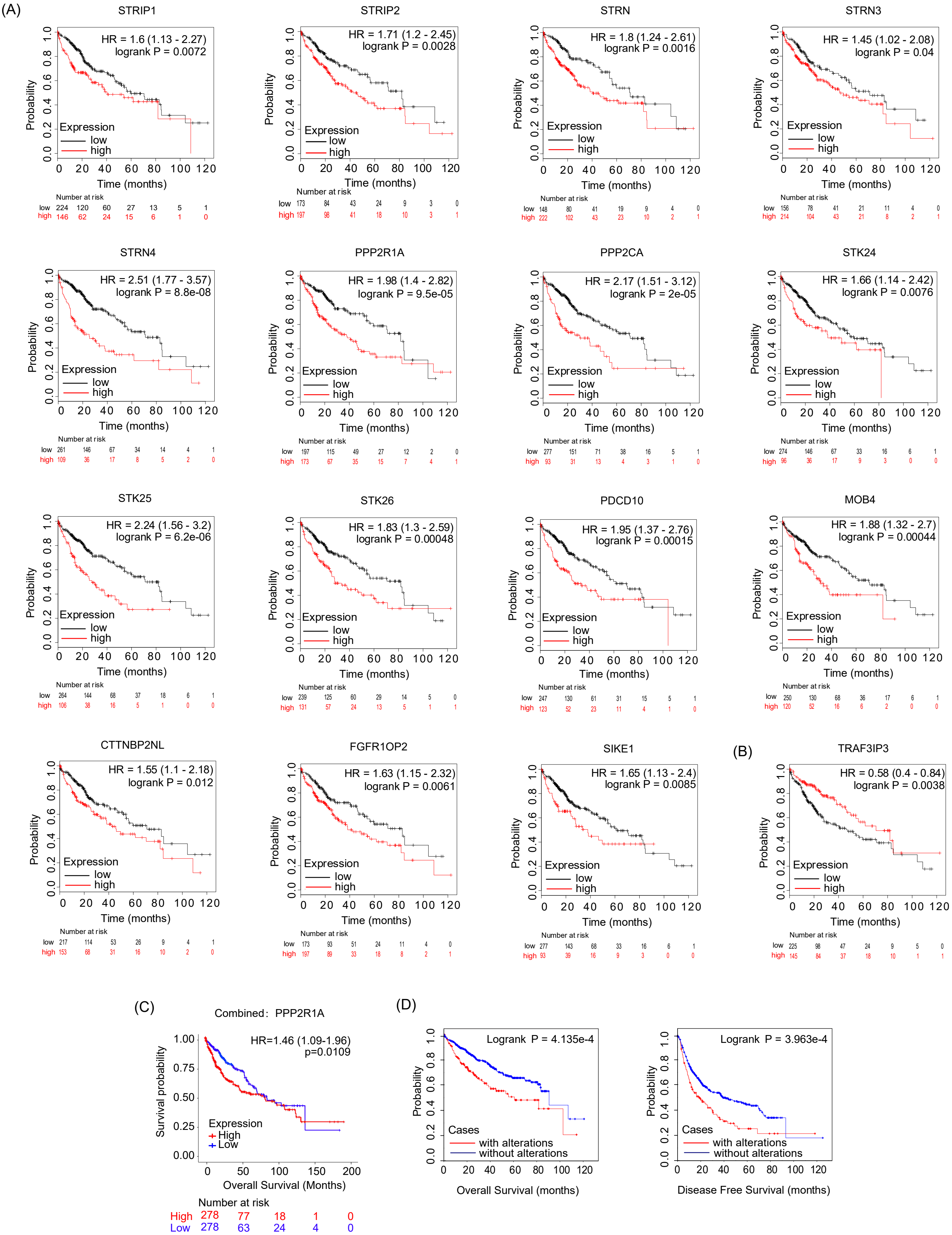

Supplymentary Figure 2. Kaplan-Meier survial plots of STRIPAK genes in liver hepatocellular carcinoma (LIHC).
