# Supplementary material for: The Dysregulation and Prognostic Analysis of STRIPAK Complex Across Cancers
Source: Front Cell Dev Biol. 2020 Jul 10;8:625. doi: 10.3389/fcell.2020.00625 (PMC7365848; doi:10.3389/fcell.2020.00625)
Supplement: TABLE S1 — STRIPAK components in Homo sapiens. [file Table_1.docx]

**Supplementary Table 1. STRIPAK components in *Homo sapiens*.**

| Protein name | Description | Gene ID in NCBI |
| --- | --- | --- |
| PPP2R1A | Protein phosphatase 2 scaffold subunit Aalpha; PP2AA | 5518 |
| PPP2CA | Protein phosphatase 2 catalytic subunit alpha; PP2AC | 5515 |
| STRIP1 | Striatin interacting protein 1 | 85369 |
| STRIP2 | Striatin interacting protein 2 | 57464 |
| STRN | Striatin; STRN1 | 6801 |
| STRN3 | Striatin 3 | 29966 |
| STRN4 | Striatin 4 | 29888 |
| SLMAP | Sarcolemma associated protein | 7871 |
| MOB4 | MOB family member 4, phocein | 25843 |
| PDCD10 | Programmed cell death 10; Cerebral cavernous malformation 3 (CCM3) | 11235 |
| STK24 | Serine/threonine kinase 24; Mammalian sterile-20-like kinase 3(MST3) | 8428 |
| STK25 | Serine/threonine kinase 25; Ste20/Oxidant Stress Response Kinase 1 (SOK1); YSK1 | 10494 |
| STK26 | Serine/threonine kinase 26; mammalian sterile-20-like kinase 4 (MST4) | 51765 |
| CTTNBP2 | Cortactin binding protein 2 | 83992 |
| CTTNBP2NL | CTTNBP2 N-terminal like | 55917 |
| TRAF3IP3 | TRAF3 interacting protein 3 | 80342 |
| FGFR1OP2 | FGFR1 (Fibroblast Growth Factor Receptor 1) Oncogene Partner 2 | 26127 |
| SIKE1 | Suppressor of IKBKE 1 | 80143 |
