# Supplementary material for: The Dysregulation and Prognostic Analysis of STRIPAK Complex Across Cancers
Source: Front Cell Dev Biol. 2020 Jul 10;8:625. doi: 10.3389/fcell.2020.00625 (PMC7365848; doi:10.3389/fcell.2020.00625)
Supplement: TABLE S3 — The prognostic information of the 18 genes of the STRIPAK complex in 21 types of cancer in Kaplan-Meier plotter. [file Table_3.docx]

**Supplementary Table 3. The prognostic information of the 18 genes of the STRIPAK complex in 21 types of cancer in Kaplan-Meier plotter.**

| Cancer Type | Numbers of samples | Overall Survival  Significant genes |
| --- | --- | --- |
| Liver hepatocellular carcinoma | 371 | 16 |
| Kidney renal clear cell carcinoma | 530 | 15 |
| lung adenocarcinoma | 513 | 13 |
| pancreatic ductal adenocarcinoma | 177 | 11 |
| Thymoma | 119 | 11 |
| Sarcoma | 259 | 9 |
| Uterine corpus endometrial carcinoma | 543 | 9 |
| Kidney renal papillary cell carcinoma | 288 | 8 |
| Bladder carcinoma | 405 | 7 |
| Head and neck squamous cell carcinoma | 500 | 7 |
| Rectum adenocarcinoma | 165 | 7 |
| Breast cancer | 1090 | 6 |
| lung squamous cell carcinoma | 501 | 6 |
| Thyroid carcinoma | 502 | 6 |
| Pheochromocytoma and Paraganglioma | 178 | 5 |
| Esophageal adenocarcinoma | 80 | 4 |
| Esophageal squamous cell carcinoma | 81 | 4 |
| ovarian cancer | 374 | 3 |
| Stomach adenocarcinoma | 375 | 3 |
| Testicular Germ Cell Tumor | 134 | 3 |
| Cervical squamou cell carcinoma | 304 | 2 |
